# The Burden of Cervical Conization in Privately Insured Young and Mid-Adult Women in the United States

**DOI:** 10.3390/vaccines11040804

**Published:** 2023-04-05

**Authors:** Kunal Saxena, Baanie Sawhney, Soham Yande, Niranjan Kathe, Sagnik Chatterjee

**Affiliations:** 1 Merck & Co., Inc., Rahway, NJ 19454, USA; 2 Complete HEOR Solutions (CHEORS), North Wales, PA 19454, USA; 3 Department of Pharmaceutical Health Outcomes and Policy, University of Houston, Houston, TX 77204, USA

**Keywords:** HPV, vaccination, conization, health-care costs burden, LEEP, CKC

## Abstract

In 2019, the United States (US) Advisory Committee on Immunization Practices (ACIP) recommended that healthcare providers engage in shared clinical decision making for adults aged 27–45 who may benefit from HPV vaccination. However, it is difficult to assess these benefits as there is a lack of data on HPV burden on young and mid-adult women. This analysis estimates the incidence of conization and the burden associated with treating pre-cancerous states related to HPV with a loop electrosurgical excision procedure (LEEP) or a cold knife conization (CKC) among commercially insured women aged 18–45. This retrospective cohort study used the IBM MarketScan commercial claims encounter database for women aged 18–45 treated with conization. We assessed the annual incidence of conization (2016–2019) and adjusted the two-year health care costs post-conization using a multivariable Generalized Linear Model (GLM)—accounting for follow-up time and other characteristics—stratified by the age groups, namely 18–26 and 27–45. The inclusion criteria were met by 6735 women, with a mean age of 33.9 years (SD = 6.2). Conization incidence was lowest for women aged 18–26 (41/100,000 to 62/100,000 women-years) and highest for women aged 31–35 (243/100,000 to 269/100,000). The GLM-adjusted, all-cause healthcare costs per patient per year were USD 7279 and USD 9249 in the 18–26 and 27–45 age groups, respectively. The adjusted costs for disease-specific care were USD 3609 and USD 4557 for women ages 18–26 and 27–45, respectively. The burden of conization and the associated costs were significant, indicating a potential healthcare benefit of HPV vaccination among young and middle-aged women.

## 1. Introduction

Human Papillomavirus (HPV) is a group of more than 150 related viruses and causes over 90% of all cervical cancers [1,2]. HPV is a sexually transmitted infection that is mostly asymptomatic and clears spontaneously, while others may cause cervical lesions (cervical intraepithelial neoplasia (CIN) grades 2, 3, and adenocarcinoma in situ, collectively referred to as CIN2+) that, if left untreated, may progress to cervical cancer [3]. The 9-valent HPV vaccine (9vHPV) targets both high-risk 9 HPV strains (types 16, 18, 31, 33, 45, 52, and 58) that may cause cancer and certain low-risk strains (6 and 11) that cause genital warts [4]. The results from clinical trials demonstrated the efficacy of the 9-valent HPV vaccine in preventing CIN2+ and cervical surgeries related to vaccine-targeted HPV types [5,6]. There is emerging evidence demonstrating the high effectiveness of HPV vaccines against cervical cancer [7,8,9], CIN2+ [10,11,12], and other HPV infections [13].

The Centers for Disease Control and Prevention’s (CDC) Advisory Committee on Immunization Practices (ACIP) recommends a routine vaccination for children ages 11 or 12 years (vaccination can be started at 9). In 2014, the ACIP extended its recommendations to administer HPV catch-up vaccines for all females aged 13–26 and all males aged 13–21 years [14]. The ACIP in June 2019 recommended using the 9-valent HPV vaccine for all adults in the 27–45 age group [15] following the FDA’s approval in October 2018 [16]. The changes to the HPV vaccination guidelines are recent, and the recommendation encourages patient–clinician shared decision-making. Even though the effectiveness of the HPV vaccine in preventing invasive cervical cancer [17] and high-grade cervical lesions [18] is well established, the uptake of HPV vaccination in this specific age group has been slow. While there may have been an increase in the administration of the HPV vaccine in this age group since the ACIP recommendations, the overall rate remains very low (the vaccination rate among women in the 27–45 age group was 66.76 per 100,000 persons in 2018 and 282.27 per 100,000 persons in 2020) [19]. The emergence of the COVID-19 pandemic in late December 2019 has led to disruptions in healthcare services, including negatively impacting routine vaccinations across the population spectrum [20]. This can be one of the reasons for the slow uptake of the HPV vaccine in this age group as well. Recent studies found that for people in the 27–45 age group, the perceived likelihood of benefitting from the vaccine and provider recommendations were important factors in receiving the vaccine [21,22]. The successful implementation of the new recommendations for HPV vaccination provides an opportunity for cost savings of HPV-related treatments due to improved prevention. Thus, a discussion between healthcare providers and individuals on the potential benefits of getting the HPV vaccine, including preventing cervical lesions, could be an important factor in improving the vaccination rates in this population. To reinstate an awareness of vaccination in this mid-adult population, highlighting the benefits of vaccination becomes ever more important.

There is limited evidence of HPV burden on the mid-adult female population compared to the younger population [23], even though the median age of causal HPV infection is approximately 24 years [24]. A large proportion of these incident infections could be easily prevented by a broader vaccination coverage of adults [25]. Thus, to facilitate shared decision making, there is a need for additional evidence of the burden of HPV infections in this population.

Recent studies have generated estimates of the economic burden of HPV-related cancers [23,26]. However, there is still limited information regarding the burden of treating pre-cancerous states, especially CIN2+ treatment. The current recommended treatment for CIN2+ involves a colposcopy coupled with one of the two cervical conization procedures: (1) loop electrosurgical excision procedure (LEEP) or (2) cold knife conization (CKC) [27]. Conization is a surgical procedure that involves removing a cone-shaped piece of tissue from the cervix. This procedure is typically performed to diagnose or treat cervical abnormalities, including pre-cancerous or cancerous cells. The procedure is recommended when abnormal cells appear on a cervical biopsy or a Pap test. If not removed, cervical lesions may progress to cervical cancer over time [27]. Cervical cancer is associated with high morbidity and mortality, and, according to the American Cancer Society, in 2023 the incidence of cervical cancer will be 13,960 and 4290 deaths will occur due to cervical cancer [28]. However, cervical cancer incidence and mortality have decreased due to increased screening and vaccination efforts [17]. HPV vaccination can significantly reduce the risk of developing cervical abnormalities and, therefore, may help reduce the overall frequency of the utilization of HPV-related diagnostic and treatment procedures.

A study published in 2012 estimated the overall annual direct cost burden associated with the prevention and treatment of HPV-related diseases to be USD 8.0 billion in the US [29]. Since routine cervical cancer screenings and follow-up visits contribute most (82.3%) of the economic burden [29], a detailed assessment of the CIN2+ treatment burden, specifically conization procedures (LEEP and CKC), is critical to highlight the potential benefits associated with HPV vaccination [30]. This study aims to estimate the overall burden associated with conization (LEEP and CKC) for the treatment of CIN2+, including the direct medical care costs from a payer perspective and the likelihood of repeat conization and CIN-related visits. To our knowledge, this is the first study to calculate the burden of treating CIN2+ with conization in the US.

## 2. Materials and Methods

### 2.1. Data Source and Study Design

Data were extracted from the IBM^®^ MarketScan^®^ Commercial Claims Encounter Databases because of its geographic representativeness of the US population, the diversity of private health insurance plans, and the ability to follow individuals longitudinally across plans [31]. The database includes claims for physician visits, outpatient visits, inpatient hospital stays, diagnostic tests, surgical procedures, and dispensed prescriptions. The data source also captures complete payment information covered by the health insurance plans and patients. A retrospective cohort study was conducted among women 18–45 years of age between 1 January 2016 and 30 June 2020 who had claims for either LEEP or CKC (Supplementary Figure S1). Conization was defined as the documentation of Current Procedure Terminology (CPT) codes for CKC (57520) or LEEP (57522) in either an inpatient or outpatient setting (diagnoses and procedure codes are provided in the Supplementary Materials).

### 2.2. Annual Incidence of Conization

The computed annual incidence rate of conization for two patient populations comprised (1) the complete population of women 18-45 years of age and (2) the subpopulation of women who received screening for HPV, defined as having a claim for an HPV test, a Pap test, or a colposcopy during the same calendar year. We assessed the incidence of conization among women screened during the same year of their conization procedure to understand better how changing clinical practices over time could impact estimates of conization incidence [11,12]

The date of the conization procedure served as the index date. Patients were required to have at least 6 months of continuous enrollment during the same calendar year as the index date and were excluded if they had any claim for pregnancy or delivery in that calendar year. Annual incidence rates (per 100,000 women-years) were calculated for 2016–2019 and were stratified into five age groups. The first age stratum is composed of women 18–26 years [32,33]. The age strata for women aged 27–45 are presented in smaller increments. The rates were calculated using the following formula (Formula (1)):

Formula (1): Annual incidence rate (per 100,000) of patients with conization

(1) $$\frac{\mathrm{Number}\;\mathrm{of}\;\mathrm{women}\;\mathrm{aged}\;18–45\;\mathrm{years}\;\mathrm{with}\;\mathrm{at}\;\mathrm{least}\;\mathrm{one}\;\mathrm{claim}\;\mathrm{for}\;\mathrm{cervical}\;\mathrm{conization}\;\mathrm{in}\;\mathrm{the}\;\mathrm{calendar}\;\mathrm{year}}{\mathrm{Total}\;\mathrm{number}\;\mathrm{of}\;\mathrm{women}\;\mathrm{aged}\;18–45\;\mathrm{years}\;\mathrm{during}\;\mathrm{the}\;\mathrm{calendar}\;\mathrm{year}\;\mathrm{in}\;\mathrm{the}\;\mathrm{database}\;}\;\times \;100,000$$

### 2.3. Healthcare Costs Associated with Conization

The cohort of women with any conization (CKC and LEEP) claim was studied for the assessment of the economic burden of treating CIN2+ with conization [27]. The index date was defined as the date of the first conization procedure that occurred between 1 July 2016 and 30 June 2018 to ensure a maximum follow-up of two years as the database extended until June 2020. The patients were followed until either the end of continuous enrollment or the completion of two years of follow-up, whichever came first. Eligibility criteria for the conization healthcare cohort included (1) 18–45 years of age at the index date; (2) at least 6 months of continuous enrollment before and after the index date; (3) at least one inpatient or outpatient claim for CKC or LEEP during the index period; and (4) the absence of claims for pregnancy or delivery, diagnoses of any cancer (including prevalent, [i.e., previously diagnosed] cervical cancer), HIV diagnosis or treatment, or other immunocompromising conditions.

The analysis extracted the health care costs associated with the first conization procedure through the complete follow-up and the 6-month follow-up after the index date. The total direct health care costs related to the index conization included medical and pharmacy costs. All-cause medical costs included inpatient admissions, emergency room visits, outpatient visits, hospice visits, and physician visits. Disease-specific and conization-specific medical costs were calculated by selecting medical claims for post-operative complications (hemorrhage, vaginal bleeding/discharge, unspecified fever, post-procedural pain, scarring of the cervix, and dysmenorrhea) and follow-up Pap tests, HPV tests, and colposcopies corresponding to ICD-10-CM, CPT, and HCPCS codes (given in Supplementary Materials). The disease-specific pharmacy costs were estimated to be the same as the all-cause pharmacy costs, as there were no NDC codes to identify the disease-specific pharmacy utilization. Costs were inflated to the 2020 US dollar based on the medical component of the consumer price index [34]. The economic burden was estimated for the complete cohort and stratified by two age groups (18–26 and 27–45).

### 2.4. Repeat Conization/CIN-Related Claims

The proportion of patients with repeat conization and claims for CIN-related visits in the 6-month follow-up period were reported to understand the burden of conization due to increased risk of residual/recurrent disease found at subsequent follow-ups.

### 2.5. Statistical Analyses

Descriptive statistics were generated to characterize the patient population (frequencies and percentages for categorical variables and measures of central tendency for continuous variables) from claims data extracted during the baseline period. Costs were reported as Per Person Per Month (PPPM) and Per Person Per Year (PPPY) to account for the cohort’s heterogeneity of follow-up time.

The adjusted costs were estimated with a multivariable Generalized Linear Model (GLM) with a gamma distribution and log link function. The gamma model accounted for the right-skewed distribution of costs. The model adjusted for covariates, including age, square of age, Charlson Comorbidity Index (CCI) score [35], region, health-plan type, number of follow-up days, and type of conization procedure. The proportion of patients with repeat conizations as well as CIN-related visits was also reported.

To specifically assess the burden of conization among unvaccinated women, we also conducted a sensitivity analysis among females who were at least 27 years of age in 2006 because they were unlikely to be vaccinated when HPV vaccination was first added to the vaccine schedules. All the analyses were conducted using SAS (version 9.4).

## 3. Results

Of the 23,569 female patients with claims for CKC or LEEP procedures during the observation period, 16,717 (70.9%) were 18-45 years of age. After applying the inclusion and exclusion criteria, the final cohort consisted of 6735 patients.

The cohort’s mean age was 33.9 years of age (SD = 6.2), with a median of 34 years (Table 1). Nearly half (49.5%) of the index procedures occurred during 2017, compared to the 26.7% in 2016 and 23.8% in 2018. Around 48% of the women had the maximum enrollment (two years after the index conization procedure). The majority of the patients lived in urban localities (80.7%) and half (50.7%) lived in the southern region of the US. Over half of the women had preferred provider organization (PPO) health insurance plans (55.2%).

Only 6% of the patients had comorbidities, as the Charlson Comorbidity Index reflected. Most patients had claims for Pap tests (87%) and colposcopy (87.4%), followed by tests for HPV (62.2%). CIN1, CIN2, and CIN3 accounted for 20%, 39.3%, and 30.0% of cytology results, respectively; a total of 35.1% of women tested positive for HPV. Most of the conization procedures were LEEP (84%).

### 3.1. Annual Conization Incidence

**Figure 1:**
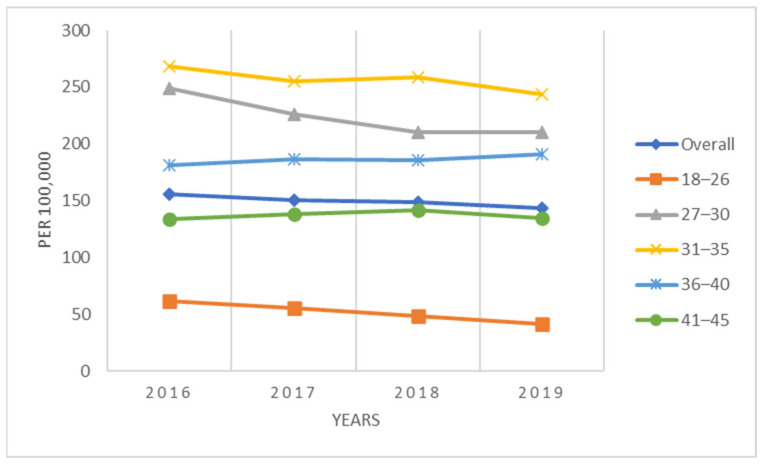
Annual incidence of conization per 100,000 women (2016–2019).

**Figure 2:**
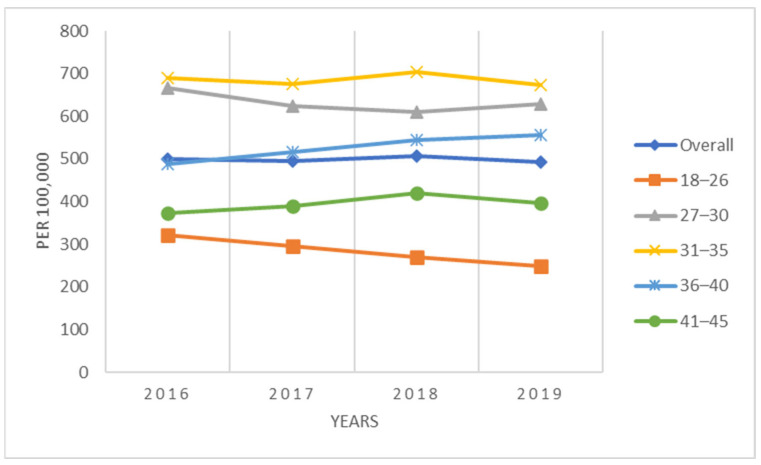
Annual incidence of conization per 100,000 screened women (2016–2019).

For the complete population of women 18–45 years of age, the annual conization incidence declined each year, starting at 156 procedures/100,000 women-years in 2016 to 143 procedures/100,000 women-years in 2019 (Figure 1). Women in the youngest age group, 18–26 years, had the lowest conization rates, ranging between 41/100,000 to 62/100,000 women-years, whereas rates for older patients were approximately 4–5 times higher. Women 31–35 years had the highest rates (243/100,000 to 269/100,000) (Figure 1). The conization rates for the sub-population of women screened for cervical cancer (i.e., HPV test, Pap test, colposcopy) during the same calendar year exhibited similar patterns but were considerably higher, ranging between 493/100,000 to 507/100,000 women-years overall (Figure 2). The conization rates were again lowest in the age group 18-26 (250–322/100,000) and highest among women ages 31–35 (673 to 705/100,000) (Figure 2).

### 3.2. Health Care Costs Associated with Conization

**Figure 3:**
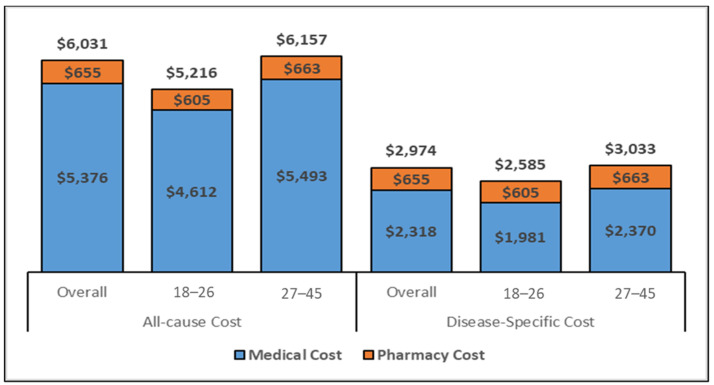
All-cause and disease-specific PPPY costs for the overall cohort (Inflated to 2020 USD) Abbreviations: USD: United States Dollar. Note. The disease-specific pharmacy costs were estimated to be same as the all-cause pharmacy costs as there were no NDC codes to identify the disease-specific pharmacy utilization.

**Figure 4:**
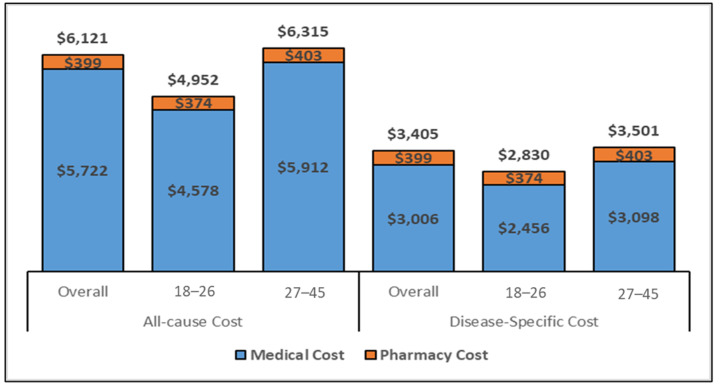
All–cause and disease-specific, 6–months costs for the overall cohort (Inflated to 2020 USD. Abbreviations: USD: United States Dollar. Note. The disease-specific pharmacy costs were estimated to be same as the all-cause pharmacy costs as there were no NDC codes to identify the disease-specific pharmacy utilization.

The estimated total medical costs associated with conization are displayed in Figure 3 and Figure 4, showcasing the medical costs (blue) and pharmacy costs (orange) for the 6-month and complete follow-up period (weighted by the follow-up time). The all-cause and disease-specific cost estimates were stratified by age (18–26 and 27–45).

The all-cause mean PPPY and PPPM costs for women aged 27–45 who underwent conization were approximately USD 1000 and USD 100 higher, respectively, as compared to the 18–26 age-group; similarly, estimated costs from the regression model were also higher for all health care and disease-specific costs during the 6-month and the complete follow-up period. Specifically, the cost PPPY (Figure 3) for the all-cause costs was USD 6031 for the overall cohort but lower (USD 5216) for younger women (ages 18–26 years) than for women 27–45 years of age (USD 6157). The pharmacy costs accounted for approximately 11% of the total costs. The PPPY costs related to disease-specific care were lower for younger women (USD 2585) than older women (USD 3033). The estimated PPPM (Supplementary Figure S2) costs exhibited similar differentials in costs by age group. Whereas the PPPM for all care was USD 432 among women ages 18–26, the PPPM for older women (aged 27–45) was USD 510. The total costs for the six months following a conization procedure (Figure 4) were comparable to the PPPY costs. The actual costs in the 6-month follow-up were higher than the PPPY costs because more costs were accumulated during the first 6 months after the procedure and the economic burden decreased over time.

### 3.3. Adjusted Total Costs based on GLM Models

The adjusted costs also displayed similar trends. The GLM-adjusted all-cause PPPY costs in the overall cohort were USD 8969, USD 7279 in the 18–26 age group, and, finally, USD 9249 in the 27–45 age group (Table 2). Further, the adjusted PPPY costs for disease-specific care were USD 4423, USD 3609, and USD 4557 for the overall cohort, 18–26, and 27–45 age group, respectively (Table 2). The all-cause and disease-specific adjusted costs in the six months were parallel to the adjusted costs in the entire time period.

### 3.4. Repeat Conization and CIN+ Diagnoses

Over the 6-month follow-up period, 29.5% (n = 1990) of the patients had at least one CIN-related visit and 4.6% repeat conization (Supplementary Table S1). Only 5.6% were diagnosed with CIN1, while 12.7% and 14.6% were diagnosed with CIN2 and CIN3.

## 4. Discussion

This study presents the rates of conization procedures and their economic burden from a commercial payer’s perspective. We found that the annual incidence of conization in the women 18–45 age group, with and without a screening of HPV, remained relatively stable over time, showing only a slight decrease between 2016 and 2019. Moreover, the findings demonstrate that the cost of conization is higher among older women (27–45 years) as compared to younger women (ages 18–26); this is in line with Stephan et al. [36].

We found that about 5% of the patients had a repeat conization procedure within 6 months, indicative of a high proportion of subsequent lesions and similar to previous studies’ findings [25,37]. An Italian retrospective analysis reported high-grade cervical lesions at 5% within two years and 6% in five years [37]. Prior research by Bogani et al. has also reported a conization recurrence rate of 4–8%, which is in line with current study estimates [37], and Reuschenbach et al. have also reported a similar rate (3%) for the German population [25]. Several reasons have been cited explaining why women remain at an increased risk of recurrence and subsequent progression to cancer: inadequate excision with positive surgical margins or persistent HPV infection, also reactivation, or HPV reinfection by exposure to an infected partner [38,39], though this paper does not capture all the reasons as our analysis is limited to the information provided by the claims data. Moreover, even though the choice of the type of conization (LEEP vs. CKC) has shown little difference in the rates of persistent or recurrent disease, [40] individual comorbidity status may have a role to play in the post-operative outcomes for women with gynecological cancers and, thus, contribute to persistent disease after the initial surgery [41].

There is limited data quantifying the economic burden of the treatment for CIN2+. Prior research has estimated the cost of CIN2+ treatment to be approximately USD 2362 (inflated to 2020 USD) per episode of care [42]; however, these estimates predate the FDA approval of HPV vaccines [32,42,43] and thus cannot be compared with the current study. Our study found that the all-cause and disease-related adjusted costs were higher in the mid-adults (ages 27–45) compared to the younger population, as also reported by Stephan et al. [36]. A recent study by Fendrick et al. estimating the economic impact of a colposcopy with a conization procedure on patients found that it is also associated with an increasing out-of-pocket cost burden of USD 1036 (in 2019 USD) [44]. Another study conducted in Korea has also emphasized that the cost of CIN2+ treatment, although lower than cervical cancer treatment cost, is nevertheless substantial [45]. A study from Singapore reported that HPV-related diseases are expected to impose a significant health and economic burden on healthcare resources in the next 25 years [46]. In summary, a considerable economic burden is associated with conization among adult women over age 25 in the United States and other high-income countries. The overall HPV-attributable economic burden is substantial in the US, much of which could be vaccine-preventable [47].

Almost a third of the women had at least one additional visit related to their initial CIN2+ diagnosis, thus emphasizing that there is a significant economic and clinical burden due to HPV-related diseases amongst all age groups; this has also been described by other studies [44,48]. Notably, the median age of causal HPV infection was estimated to be approximately 24 years, and per the HPV natural history, the average time from infection to the diagnosis of CIN or conization is 1–3 years [24,25,49,50], thus the age at CIN diagnosis would be greater than 25 years; this is validated by the HPV-IMPACT study of adult women that found the mean age at CIN2+ diagnosis was approximately 28 years [51]. Further, since not all patients with CIN2+ may be treated at diagnosis, the median age at conization would be even greater; our study found it to be 34 years.

Moreover, the CDC reports that having multiple sexual partners and acquiring a new partner increases the risk of HPV infection and cervical cancer [52,53], which is particularly common amongst the mid-adult population (i.e., the 27–45 age group) [54]. In line with this, our findings suggest that the highest proportion of incident conization procedures is in the mid-adult population. Given that half of the infections that may cause cancer can occur after approximately 24 years and the prevalence of high-risk factors in mid-adults, the burden associated with the disease could be easily prevented not only by vaccinating adolescents but also the high-risk mid-adult population [24,55]. Even though the most effective measure for preventing HPV-associated diseases (such as CIN2+and cervical cancer) is prophylactic HPV vaccination [25], the cumulative vaccination rates of adults aged 18−26 years have remained at relatively low levels in the US (i.e., 35%) [26].

This study has limitations inherent in claims databases, i.e., information and misclassification bias related to medical coding. The findings might also underestimate the incidence and the burden of conization because the study only captured services for which an insurance claim was generated. The study population was restricted to commercially insured women and not generalizable to the uninsured and those covered by federal health insurance [56]. While we aimed to use a nationally representative database to estimate the cost of conization and follow patients over two years, we did not assess regional differences in practices or changes over time in the US. Future studies can be conducted to evaluate whether variations in treatment protocols or surveillance exist within the US and their impact on the financial burden of patients undergoing conization.

The follow-up time of 2 years to investigate the successive CIN diagnoses and conizations after the initial conization is relatively short, considering the average duration of CIN2+ development is 1–3 years [37,57].

Furthermore, the study only included adult women aged 18–45 years as the primary disease burden was expected to be the highest in this age group, with women being sexually active and of childbearing age. Further studies are needed to explore the disease burden on other age groups.

An additional limitation was that the HPV vaccination status of the women included in this study was unknown. It is difficult to determine whether women in our cohort were vaccinated based on the medical claims data. Assessing vaccination status may require tracking patients’ medical history over a long period of time since some adults may have been vaccinated as adolescents or young adults. Following individuals longitudinally across decades requires them to be continuously enrolled in an insurance plan with the same employer for the entire duration. However, this is hardly the case and thus presents a limitation inherent to database studies—the lack of complete medical histories [58]. Our study would have greatly benefitted from the information on the HPV vaccination status and, consequently, the vaccine’s impact on the conization burden. However, even in the absence of this information, our study offers a perspective that can serve as a basis for future assessments of the impact of vaccines on resource consumption using prospective data.

## 5. Conclusions

Conization procedures are a source of substantial economic burden on all populations, irrespective of age, in the US and are markedly higher among women 27–45 years of age. Compared to women aged 18–26, women in the 27–45 age group have more conization procedures annually and have a higher economic burden. Mid-adult HPV vaccination has the potential to preserve women’s health and reduce future healthcare costs.

## Figures and Tables

**Table 1:** Baseline Characteristics.

Metric	Cohort [N = 6735]
Age at index-date (years)	
Mean± SD [Median] (Min, Max)	33.9 ± 6.2 [34.0] [18.0, 45.0]
Age-group at Index-date [N (%)]	
18–26	958 (14.2)
27–30	1211 (18.0)
31–35	1818 (27.0)
36–40	1518 (22.5)
41–45	1230 (18.3)
Index-year [N (%)]	
2016	1798 (26.7)
2017	3333 (49.5)
2018	1604 (23.8)
Follow-up days	
Mean± SD [Median] (Min, Max)	542.4 ± 213.8 [681.0] [180.0, 730.0]
Proportion of patients with full 2 years of follow-up [N (%)]	
Follow-up	3231 (48.0)
Urbanicity [N (%)]	
Urban	5433 (80.7)
Rural	1302 (19.3)
Geographic Region [N (%)]	
Northeast	1073 (15.9)
North Central	1278 (19.0)
South	3417 (50.7)
West	957 (14.2)
Others and Unknown	10 (0.2)
Health Plan Type [N (%)]	
Comprehensive	128 (1.9)
EPO	56 (0.8)
HMO	687 (10.2)
POS	484 (7.2)
PPO	3716 (55.2)
POS with capitation	116 (1.7)
CDHP	745 (11.1)
HDHP	672 (10.0)
Unknown	131 (1.9)
Charlson Comorbidity Index (CCI)	
Mean± SD [Median] (Min, Max)	0.1 ± 0.3 [0.0] {0.0, 4.0}
CCI Categories [N (%)]	
0	6321 (93.9)
1 to 2	405 (6.0)
≥3	9 (0.1)
Comorbidities [N (%)]	
CIN1	1345 (20.0)
CIN2	2644 (39.3)
CIN3	2061 (30.0)
HPV infection	2367 (35.1)
Cervical Cancer	0 (0.00)
Laboratory Tests [N (%)]	
HPV Test	4192 (62.2)
Pap Test	5858 (87.0)
Colposcopy	5887 (87.4)
Type of conization procedure (CKC, LEEP) [N (%)]	
CKC	1078 (16.0)
LEEP	5657 (84.0)

Abbreviations: CCI: Charlson Comorbidity Index; CDHP: Consumer-Driven Health Plan; CKC: Cold Knife Conization; EPO: Exclusive Provider Organization; HDHP: High Deductible Health Plan; HMO: Health Maintenance Organization; LEEP: Loop Electrosurgical Excision Procedure; Max: Maximum; Min: Minimum; POS: Point of Service; PPO: Preferred Provider Organization; SD: Standard Deviation.

**Table 2:** All-cause and disease-specific adjusted total costs based on GLM models.

Label	Complete Follow-Up	6-Month Follow-Up
All-cause: Mean (SD)		
Overall	USD 8969.2 (2,769.1)	USD 6119.9 (1,900.9)
Age 18–26	USD 7278.7 (1,914.3)	USD 4952.2 (1,227.8)
Age 27–45	USD 9248.8 (2,819.3)	USD 6314.7 (1,958.5)
Disease-specific: Mean (SD)		
Overall	USD 4423.4 (1,366.2)	USD 3407.7 (956.9)
Age 18–26	USD 3609.1 (1,035.2)	USD 2835.6 (771.7)
Age 27–45	USD 4557.3 (1,396.0)	USD 3503.6 (993.9)

## Data Availability

The original de-identified data used in this analysis were obtained from and are the property of IBM MarketScan Databases. IBM MarketScan has restrictions prohibiting the authors from making the data set publicly available. Interested researchers may contact IBM MarketScan Databases to apply to gain access to the study’s data.

## Supplementary Materials

The following supporting information can be downloaded at: https://www.mdpi.com/article/10.3390/vaccines11040804/s1, Figure S1: Study Design for estimating cost and HCRU of conization; Figure S2: PPPM costs for the overall cohort (Inflated to 2020 USD); Table S1: Rate of repeat conization and CIN diagnosis in 6-months’ follow-up period; Table S2: HCRUs in complete/6 months’ time period; Table S3: ICD-10 diagnosis codes for pregnancy, delivery; Table S4: ICD-10-PCS and CPT4 procedure codes for cervical conization; Table S5: CPT//ICD-10-CM Codes for Post-Operative Complication/Adverse Event/Secondary Procedures after conization; Table S6: ICD-9-CM/ICD-10-CM Codes for Comorbidities; Table S7: CPT/ICD procedure codes for Laboratory Tests; Table S8: CPT 4 Procedure codes for HPV vaccine; Table S9: ICD-9-CM/ICD-10-CM Codes for Exclusion criteria: All Cancers/HIV/Immunosuppressive conditions; Table S10: Drugs for Immunosuppressive conditions.
