# Supplementary material for: The Burden of Cervical Conization in Privately Insured Young and Mid-Adult Women in the United States
Source: Vaccines (Basel). 2023 Apr 5;11(4):804. doi: 10.3390/vaccines11040804 (PMC10142968; doi:10.3390/vaccines11040804)
Supplement: Supplementary file 1 [file vaccines-11-00804-s001.zip › vaccines-2234219-supplementary.pdf]

Supplementary Materials

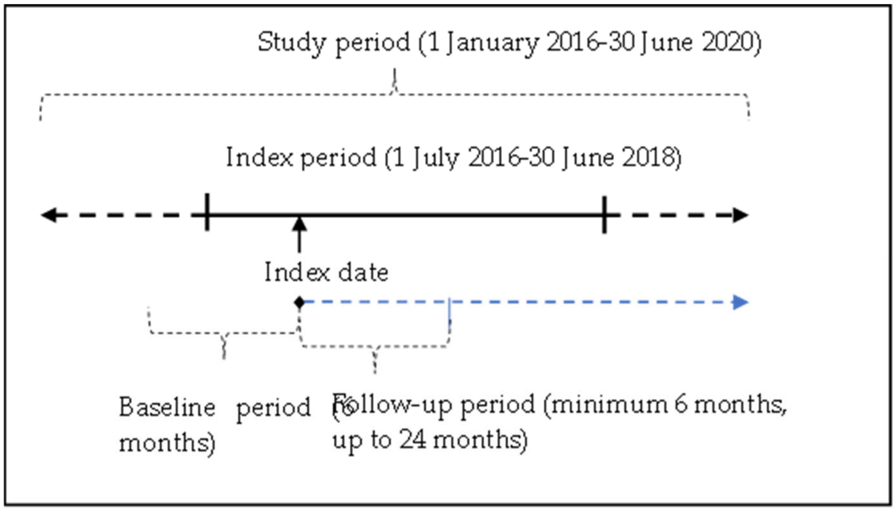

Figure S1. Study Design for estimating cost and HCRU of conization.

The figure uses a timeline diagram to describe the elements of the study design. The study period ranged from 01 January 2006 to 30 June 2020. The index period (patient identification period) ranges from 1 July 2016 to 30 June 2018. The index date was defined as the first evidence of conization within the study period. Patients were required to have a baseline period of 6 months and a follow-up period of up to 2 years with a minimum of 6 months requirement.

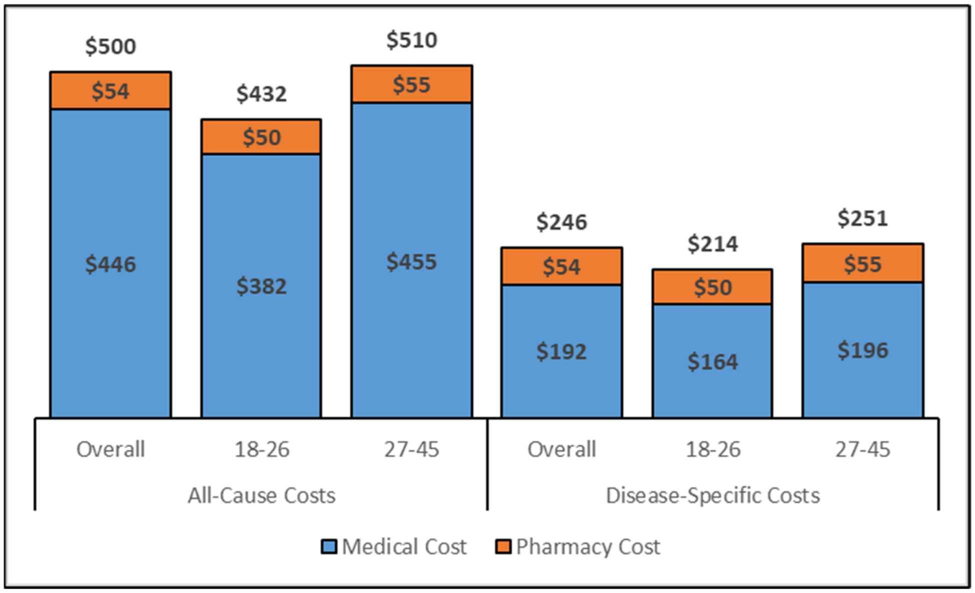

Figure S2. PPPM costs for the overall cohort (Inflated to 2020 USD).Abbreviations: USD: United States Dollar. Note. The disease-specific pharmacy costs were estimated to be the same as the all-cause pharmacy costs, as there were no NDC codes to identify the disease-specific pharmacy utilization.

Table S1. Rate of repeat conization and CIN diagnosis in 6-months' follow-up period.

Frequency	Overall Cohort (N=6,735)
**Rate of repeat Conization**	
**0**	6,427 (95.43)
**1**	301 (4.47)
**2**	6 (0.09)
**3**	1 (0.01)
**Rate of repeat CIN diagnosis**	
**0**	4,745 (70.45)
**1**	1,480 (21.97)
**2**	329 (4.88)
**>=3**	181 (2.7)
**Rate of repeat CIN1 diagnosis**	
**0**	6,361 (94.5)
**1**	331 (4.9)
**2**	36 (0.5)
**>=3**	7 (0.1)
**Rate of repeat CIN2 diagnosis**	
**0**	5,874 (87.2)
**1**	713 (10.6)
**2**	109 (1.6)
**>=3**	30 (0.6)
**Rate of repeat CIN3 diagnosis**	
**0**	5,703 (84.7)
**1**	728 (10.8)
**2**	183 (2.7)
**>=3**	73 (1.8)

Table S2. HCRUs in complete/6 months' time period.

HCRU	Overall Cohort - Mean (SD)		18–26 age-group - Mean (SD)		18–26 age-group - Mean (SD)	
	All-cause	Disease Specific	All-cause	Disease Specific	All-cause	Disease Specific
**PPPY HCRUs in complete time period**						
**ER Visits**	0.23 (0.72)	0.02 (0.17)	0.30 (0.80)	0.03 (0.21)	0.22 (0.71)	0.02 (0.16)
**Hospice Visits**	0.00 (0.07)	0.00 (0.01)	0.00 (0.00)	0.00 (0.00)	0.00 (0.08)	0.00 (0.01)
**Outpatient Visits**	5.01 (4.25)	1.29 (1.40)	5.02 (4.07)	1.43 (1.42)	5.01 (4.28)	1.27 (1.40)
**Physician Visits**	2.74 (4.79)	0.61 (0.83)	2.44 (3.84)	0.68 (0.90)	2.78 (4.92)	0.59 (0.82)
**Other Visits**	2.23 (2.93)	0.89 (1.17)	2.37 (2.89)	1.05 (1.24)	2.37 (2.89)	0.87 (1.15)
**Pharmacy Visits**	6.45 (7.51)	6.45 (7.51)	6.86 (7.19)	6.86 (7.19)	6.38 (7.56)	6.38 (7.56)
**HCRUs in 6-months' time period**						
**ER Visits**	0.18 (0.63)	0.02 (0.18)	0.20 (0.68)	0.03 (0.22)	0.17 (0.62)	0.02 (0.17)
**Hospice Visits**	0.00 (0.09)	0.00 (0.01)	0.00 (0.00)	0.00 (0.00)	0.00 (0.09)	0.00 (0.01)
**Outpatient Visits**	3.73 (3.11)	1.30 (1.36)	3.51 (2.72)	1.30 (1.26)	3.77 (3.17)	1.30 (1.37)
**Physician Visits**	1.94 (3.18)	0.55 (0.78)	1.73 (2.79)	0.59 (0.76)	1.97 (3.24)	0.55 (0.79)

**Other Visits**	1.67 (2.38)	0.73 (1.12)	1.68 (2.08)	0.83 (1.18)	1.67 (2.42)	0.71 (1.11)
**Pharmacy Visits**	4.27 (4.65)	4.27 (4.65)	4.41 (4.40)	4.41 (4.40)	4.25 (4.69)	4.25 (4.69)

Abbreviations: HCRU: Healthcare Resource Utilization. SD: standard deviation. ER: Emergency Room. PPPY: Per patient per year.The table represents the mean utilization of the following components of healthcare resources: Emergency room visits, Hospice visits, Outpatient visits, physician visits, pharmacy visits, and other visits for the study sample in the 2 years following index conization (represented as PPPY utilization) and the 6 months following index conization.

Table S3. ICD-10 diagnosis codes for pregnancy, delivery.

ICD-10	Description
O60-O77	Complications of labor and delivery
O80-O82	Encounter for delivery
O60-O77	Complications of labor and delivery
O00-O09	Pregnancy with abortive outcome

Table S4. ICD-10-PCS and CPT4 procedure codes for cervical conization:.

Description	CPT4	ICD-10-PCS
Cold Knife Cervical Conization	57520	
Loop Excision Cervical Conization	57522	
Excision of Cervix, Via Natural or Artificial Opening		0UBC7ZZ

Table S5. CPT//ICD-10-CM Codes for Post-Operative Complication/Adverse Event/ Secondary Procedures after conization.

Post-Operative Complication / Adverse Event	ICD-10-CMS
Hemorrhage	R58
Vaginal bleeding	N93.8
Vaginal Discharge	N89.8
Febrile body temperature/ Fever	R50.9
Pain	
Mild cramping and discomfort (abdominal pain)	R10.10, R10.2, R10.30
Scarring of the cervix	N88.1
Menstrual-like cramps (Dysmenorrhea)	N94.6
CIN1/2/3	N87.0<br>N87.1<br>D06.0

	D06.0<br>D06.1<br>D06.7
Abnormal Pap	R87610-R87619
**Secondary Procedures after conization**	**ICD-10 PCS/ CPT Codes**
Conization (CKC/LEEP)	0UBC7ZZ
Other Procedures on the cervix (Includes Pap test, Biopsy of cervix, Electrocautery of cervix, Thermal cautery of cervix, Cryocautery of cervix, Laser ablation of cervix, Conization of cervix using cold knife, Excision of cervix using loop electrode, Amputation of cervix, Colposcopy, Cerclage of uterine cervix, Plastic repair of uterine cervix by vaginal approach)	57510, 57511, 57513 Other surgical cautery of cervix<br>57530-57531 Trachelectomy (amputation of cervix) 57540, 57545, 57550, 57555, 57556 Excision of cervical stump
Hysterectomy	51925, 56308, 58150, 58152, 58200, 58210, 58240, 58260, 58262, 58263, 58267, 58270, 58275, 58280, 58285, 58290-58294, 59525, 58548, 58550, 58552- 58554, 58570-58573, 58951, 58953, 58954, 58956, 59135
Pap test	•88175<br>•Z01.42 (ICD-10)<br>•88141, 88142, 88143, 88147, 88147, 88150, 88152, 88153, 88154, 88164, 88165, 88166, 88167, 88174, 88175<br>•G0123, G0124,<br>G0141, G0143, G0144, G0145, G0147, G0148, P3000, P3001, Q0091
HPV test	•87623<br>•87624<br>•87625<br>•87620, 87621, 87622
Colposcopy	•57420<br>•57421<br>•57452<br>•57454<br>•57455

	•57456<br>•57460<br>•57461	

Table S6. ICD-9-CM/ICD-10-CM Codes for Comorbidities.

Comorbidities	ICD 9-CM	ICD 10
CIN 1	• 622.11	• N87.0
CIN 2	• 622.12	• N87.1
CIN 3	• 233.1	• D06.0 Carcinoma in situ of cervix uteri<br>• D06.0 Carcinoma in situ of endocervix<br>• D06.1 Carcinoma in situ of exocervix<br>• D06.7 Carcinoma in situ of other parts of cervix<br>• D06.9 Carcinoma in situ of cervix, unspecified
HPV infection	• 795.05	• R87.810
Cervical Cancer	• 180.9	• C53.9

Table S7. CPT/ICD procedure codes for Laboratory Tests.

Test	Description	CPT/ICD
HPV	•Low risk types (eg, 6, 11, 42, 43, 44)<br>•high-risk types (eg, 16, 18, 31, 33, 35, 39, 45, 51, 52, 56, 58, 59, 68)<br>•Types 16 and 18 only, includes type 45, if performed<br>•HPV Test	•87623<br>•87624<br>•87625<br>•87620, 87621, 87622
Pap test	•Cervical Cytology	•88175<br>•V72.32 (ICD-9)<br>•Z01.42 (ICD-10)<br>•88141, 88142, 88143, 88147, 88147, 88150, 88152, 88153, 88154, 88164, 88165, 88166, 88167, 88174, 88175<br>•G0123, G0124, G0141, G0143, G0144, G0145, G0147, G0148, P3000, P3001, Q0091
Colposcopy	•Colposcopy of the entire vagina, with cervix if present<br>•Colposcopy of the entire vagina, with cervix if present; with biopsy(s)<br>•Colposcopy of the cervix including upper/adjacent vagina<br>•Colposcopy of the cervix including upper/adjacent vagina; with biopsy(s) of the cervix and endocervical curettage<br>•Colposcopy of the cervix including upper/adjacent vagina; with biopsy(s) of the cervix<br>•Colposcopy of the cervix including upper/adjacent vagina; with endocervical curettage	•57420<br>•57421<br>•57452<br>•57454<br>•57455<br>•57456

•Colposcopy of the cervix including upper/adjacent vagina; with loop electrode biopsy(s) of the cervix	•57460
•Colposcopy of the cervix including upper/adjacent vagina; with loop electrode conization of the cervix	•57461
•Vaginoscopy	•70.12 (ICD-9 proc)

Table S8. CPT 4 Procedure codes for HPV vaccine.

Vaccine	CPT4
GARDASIL	90649
CERVARIX	90650
GARDASIL 9	90651

Table 9. ICD-9-CM/ICD-10-CM Codes for Exclusion criteria: All Cancers / HIV / Immunosuppressive conditions.

Description	ICD- 9 Code	ICD-10 Code		
**Human immunodeficiency virus (HIV)**	**042, V08, 079.53**	B20-B24, Z21, B97.35		
Cancer	140-209, 230-234	C00-C95, C7A D00-D09		
Hematologic malignancy (HM)	200.xx-208.92 238.4x, 238.6x	D49.4 D47.Z9		
Disorders involving the immune mechanism	279.xx	D80-D89		
Aplastic anemia and other bone marrow failure syndromes	284.xx	D61.810, D61.811, D61.818 D61.82		
**Procedures**	**ICD-9 dx/proc**	**ICD-10 dx/proc**	**CPT**	**HCPCS**
Organ transplantation (including hematopoietic stem cell transplant (HCT))	199.2x, 238.77, 414.06, 414.07, 996.8x, E878.0, V42.xx, V45.87, V58.44, 00.91-00.93, 07.94, 11.60, 11.69, 33.50-33.6x, 37.51, 41.0x, 46.97, 50.4x, 50.5x, 52.80, 52.83,	C80.2, D47.Z1, I25.811, I25.812, T86.9x, T86.1x, T86.4x, T86.2x, T86.8x, T86.0x, T86.5x Y83.0, Z94.xx, Z95.3 Z98.85 Z48.298, 0xYxxxx	00144, 00580, 00796, 00868, 29868, 32851-32856, 33933-33935, 33944-33945, 38240, 38241, 38242, 44135-44137, 44715-44721, 47135-47136, 47143-47147, 48551-48556, 50323-50370, 65710-65757, 65780-65781, 76776-76778, 81267-81268	G0341-G0343, G0369, Q0510, S2052-S2054, S2060, S2065, S2102-S2103, S2109, S2142-S2152, S9085

	52.85, 52.86, 55.53, 55.69			
Procedures indicating: Injection or infusion of cancer chemotherapeutic substance	528.01, 960.7x, 963.1x, 999.81, E930.7, E933.1, V58.11, V66.2x, V67.2x	K12.31, K12.33 T45.1X1A T451X1A, T451X2A, T451X3A, T451X4A, T80810A, Z5111 Z5189 Z08, Z09	36260, 36640, 96401, 96402, 96409, 96411, 96413, 96415, 96416, 96417, 96420, 96422, 96423, 96425, 96440, 96445, 96549	C8953-C8955, S9329- S9331, Q0083, Q0084, Q0085, S1016
Procedures indicating: Radiation therapy	V58.0x, V66.1x, V67.1x, 17.61, 92.2x, 92.30, 92.31, 92.32, 92.33, 92.39, 92.41, 99.85	Z510, Z5189, Z08, Z09, Dxxxxxx	32998, 50559, 50578, 50959, 50978, 52250, 58346, 61796, 61797, 61798, 61799, 63620, 63621, 77371, 77372, 77373, 77380, 77381, 77401, 77402, 77403, 77404, 77406, 77407, 77408, 77409, 77411, 77412, 77413, 77414, 77416, 77418, 77422, 77423, 77424, 77425, 77427, 77431, 77432, 77435, 77469, 77470, 77499, 77520, 77522, 77523, 77525, 77600, 77605, 77610, 77615, 77620, 77750, 77761, 77762, 77763, 77776, 77777, 77778, 77781, 77782, 77783, 77784, 77785, 77786, 77787, 77789, 79005, 79030, 79035, 79100, 79101, 79200, 79300, 79400, 79403, 79420, 79440, 79445, 79999, 0082T, 0083T, 0182T	A9523, A9534, A9699, C1081, C1083, C1715, C1716, C1717, C1718, C1719, C1720, C2616, C2633, C2634, C2635, C2636, C9401, C9402, C9405, G0173, G0242, G0243, G0251, G0339, G0340, Q3001, S8049

Table S10. Drugs for Immunosuppressive conditions.

Treatment with:

- Chemotherapy
- Radiation therapy
- TNF inhibitors
- Protease inhibitors
- Reverse transcriptase inhibitors
- Azathioprine, cyclosporine, or tacrolimus

Drug Name	HCPCS
Abatacept	J0129
Adalimumab	J0135
Aldesleukin	J9015
Alemtuzumab	J9010
Alimta (LY231514)	
Altretamine	
Aminoglutethimide	
Anakinra	
Antithymocyte Globulin	J7504
Arsenic trioxide	J9017
Asparaginase	J9020
Auranofin	
Azacitidine	J9025
Azathioprine	J7500, J7501
Basiliximab	J0480
BCG live vaccine	J9031
Bexarotene	
Bleomycin	J9040
Bortezomib	J9041
Busulfan	J0594, J8510
Capecitabine	J8520, J8521
Carboplatin	J9045
Carmustine	J9050
Chlorambucil	J0720, S0172
Chromic or Na P-32	
Cisplatin	C9418, J9060, J9062
Cladribine	J9065
Clofarabine	J9027
Cyclophosphamide	J8530, J9070, J9080, J9090-J9097
Cyclosporin	J7502, J7515, J7516
Cytarabine	J9098, J9100, J9110
Dacarbazine	J9130, J9140
Daclizumab	J7513
Dactinomycin	J9120
Dasatinib	

Daunorubicin	J9150, J9151
Decitabine	J0894
Denileukin deftitox	J9160
Docetaxol	J9170, J9171
Doxorubicin	J9000, J9001, J9010, C9415, Q2048, Q2049
Eculizumab	J1300
Epirubicin	J9178
Estramustine	
Etanercept	J1438
Etoposide	J8560, J9181, J9182, C9414, C9425
Floxuridine	J9200
Fludarabine	C9262, J8562, J0185, Q2025
Fluorouracil	J9190, S3722
GCSF (as a proxy)	J8560, J9181, J9182, C9414, C9425
Gemcitabine	J9201
Gemtuzumab	J9300
Hexamethylmelamine	
Hydroxyurea	S0176
Ibritumomab Tiuxetan	A9543
Idarubicin	J9211
Ifosfamide/mesna	C9427, J9208
Imatinib Mesylate	S0088
Infliximab	J1745
Interferon alfa-2A	J9213, S0145
Interferon alfa-2B	J9214, S0146, S0148
Interferon alfa-N3	
Interferon beta-1A	
Interferon beta-1B	
Interferon gamma-1B	
Irinotecan	J9206
Ixabepilone	C9240, J9207
Leflunomide	
Lenalidomide	
Lomustine	S0178
MAB-B43.13	
Mechlorethamine	J9230
Melphalan	J9245, J8600
Mercaptopurine	S0108
Methotrexate	J9250, J9260, J8610
Mitomycin	
Mitoxantrone	J9293
Muromonab-CD3	J7505
Mycophenolate mofetil	J7517
Nelarabine	J9261

Nilotinib	
Nitrogen Mustard	
Oxaliplatin	J9263, C9205
Paclitaxel	J9264, J9265, C9431
Panitumumab	J9303
Pegaspargase	J9266
Peginterferon alfa-2A	J9213, S0145
Peginterferon alfa-2B	J9214, S0146, S0148
Pemetrexed disodium	C9213, J9305
Penicillamine	
Pentostatin	J9268
Pipobroman	
Procarbazine	S0182
Rituximab	J9310
Sirolimus	J7520
Streptozocin	J9320
Tacrolimus	J7507, J7525
Temozolomide	C9253, J8700, J9328
Temsirolomus	C9239, J9330
Teniposide	Q2017
Thalidomide	
Thioguanine	
Thiotepa	J9340
TLK286	
Topotecan	J8705, J9350, J9351,
Tositumomab	A9545, G3001
Tretinoin	S0117
Trimetrexate	J3305
Vinblastine	J9360
Vincristine	J9370, J9375, J9380
Vinorelbine	J9390

- Systemic Corticosteroids

**Prednisone**	**> 7.5 mg/day**
prednisolone	> 7.5 mg/day
Hydrocortisone	> 30 mg/day
Methylprednisolone	> 6 mg/day
Dexamethazone	> 1.125 mg/day
